# A 3D-Video-Based Computerized Analysis of Social and Sexual Interactions in Rats

**DOI:** 10.1371/journal.pone.0078460

**Published:** 2013-10-30

**Authors:** Jumpei Matsumoto, Susumu Urakawa, Yusaku Takamura, Renato Malcher-Lopes, Etsuro Hori, Carlos Tomaz, Taketoshi Ono, Hisao Nishijo

**Affiliations:** 1 System Emotional Science, Graduate School of Medicine and Pharmaceutical Sciences, University of Toyama, Toyama, Japan; 2 Department of Judo Neurophysiotherapy, Graduate School of Medicine and Pharmaceutical Sciences, University of Toyama, Toyama, Japan; 3 Primate Center and Laboratory of Neurosciences and Behavior, Department of Physiological Sciences, Institute of Biology, University of Brasília, Brasilia, DF, Brazil; Cajal Institute, Consejo Superior de Investigaciones Científicas, Spain

## Abstract

A large number of studies have analyzed social and sexual interactions between rodents in relation to neural activity. Computerized video analysis has been successfully used to detect numerous behaviors quickly and objectively; however, to date only 2D video recording has been used, which cannot determine the 3D locations of animals and encounters difficulties in tracking animals when they are overlapping, e.g., when mounting. To overcome these limitations, we developed a novel 3D video analysis system for examining social and sexual interactions in rats. A 3D image was reconstructed by integrating images captured by multiple depth cameras at different viewpoints. The 3D positions of body parts of the rats were then estimated by fitting skeleton models of the rats to the 3D images using a physics-based fitting algorithm, and various behaviors were recognized based on the spatio-temporal patterns of the 3D movements of the body parts. Comparisons between the data collected by the 3D system and those by visual inspection indicated that this system could precisely estimate the 3D positions of body parts for 2 rats during social and sexual interactions with few manual interventions, and could compute the traces of the 2 animals even during mounting. We then analyzed the effects of AM-251 (a cannabinoid CB1 receptor antagonist) on male rat sexual behavior, and found that AM-251 decreased movements and trunk height before sexual behavior, but increased the duration of head-head contact during sexual behavior. These results demonstrate that the use of this 3D system in behavioral studies could open the door to new approaches for investigating the neuroscience of social and sexual behavior.

## Introduction

A large number of studies have examined the various neural functions associated with social and sexual interactions in rodents [1], [2], [3], [4], [5], [6]. Computerized video analysis allows numerous behaviors to be detected much more quickly and objectively than behavioral analysis based on visual inspection [7]. Previous computerized video analysis systems (e.g., MiceProfiler [7]; SocialScan, Cleversys; Ethovision, Noldus; VideoTrack, Viewpoint) have used 2D top-view video recording and detected behaviors as follows: first, silhouettes of the animals in the video frames were extracted based on the animals' colors; the positions of the body parts (head, trunk, etc.) of the animals were then estimated based on these silhouettes; and finally, behaviors were recognized based on the spatio-temporal patterns of these positions. However, such 2D video analyses have notable limitations. First, estimation of the positions of animals is limited to a 2D horizontal plane, which makes it difficult to recognize some behaviors that involve vertical movements (e.g., rearing). Second, when animals with the same colors overlap, e.g., during mounting, it is difficult to distinguish the animals from the overlapping silhouette. Although this problem could be avoided by painting the animals different colors, such manipulations may bias the animals' behaviors and the positions of the occluded body parts could still not be estimated (see Supplementary Notes 2 and 3 in [7] for further details of these limitations).

In the present study, we aimed to overcome these limitations by developing a novel 3D video analysis system. In this system, a 3D image was reconstructed by integrating images captured by multiple cameras from different viewpoints, and 3D positions of the body parts were then estimated by fitting a skeleton model of the animal to the 3D images. Because multiple cameras were used, occlusion was much less likely to occur in this system than in the previous 2D systems. Thus, this system enabled estimation of the 3D positions of the body parts of 2 rats during interactions and the continual tracking of these body parts even during mounting. To the best of our knowledge, no previous study has used computerized video analysis to examine the sexual behavior of rodents, probably due to the difficulties encountered in tracking positions, particularly during mounting. However, the use of 3D video analysis in the present system was expected to enable the quantitative analysis of sexual behaviors.

To test the effectiveness of this system for use in behavioral analyses, we used it to investigate the effect of cannabinoid receptor antagonists on the sexual behavior of male rats. It has previously been reported that cannabinoid receptor antagonists facilitate male sexual behavior, and thus, their use has been suggested for the treatment of sexual function disorders [8], [9]. There are many possible mechanisms by which cannabinoid receptor antagonists may function as they occur in many different parts of the brain [9]. In the present study, we used the 3D video analysis system to analyze the effects of AM-251 (a cannabinoid receptor CB1 antagonist) on male rat sexual behavior. We found that AM-251 had significant effects on male sexual behavior, which could be detected through 3D spatial measurements of the body parts.

## Materials and Methods

### Overview of the system

In previous investigations into the social behavior of rodents, video analysis systems have estimated the 2D positions of major body parts (head, trunk, etc.) of each animal and then recognized various behaviors based on these spatio-temporal patterns of the positions. In the present study, we aimed to extend this approach into 3D, i.e., to track the 3D positions of 4 body parts (head, neck, trunk, and hip) of each rat and to recognize behaviors based on the spatio-temporal patterns of the 3D positions.

**Figure 1:**
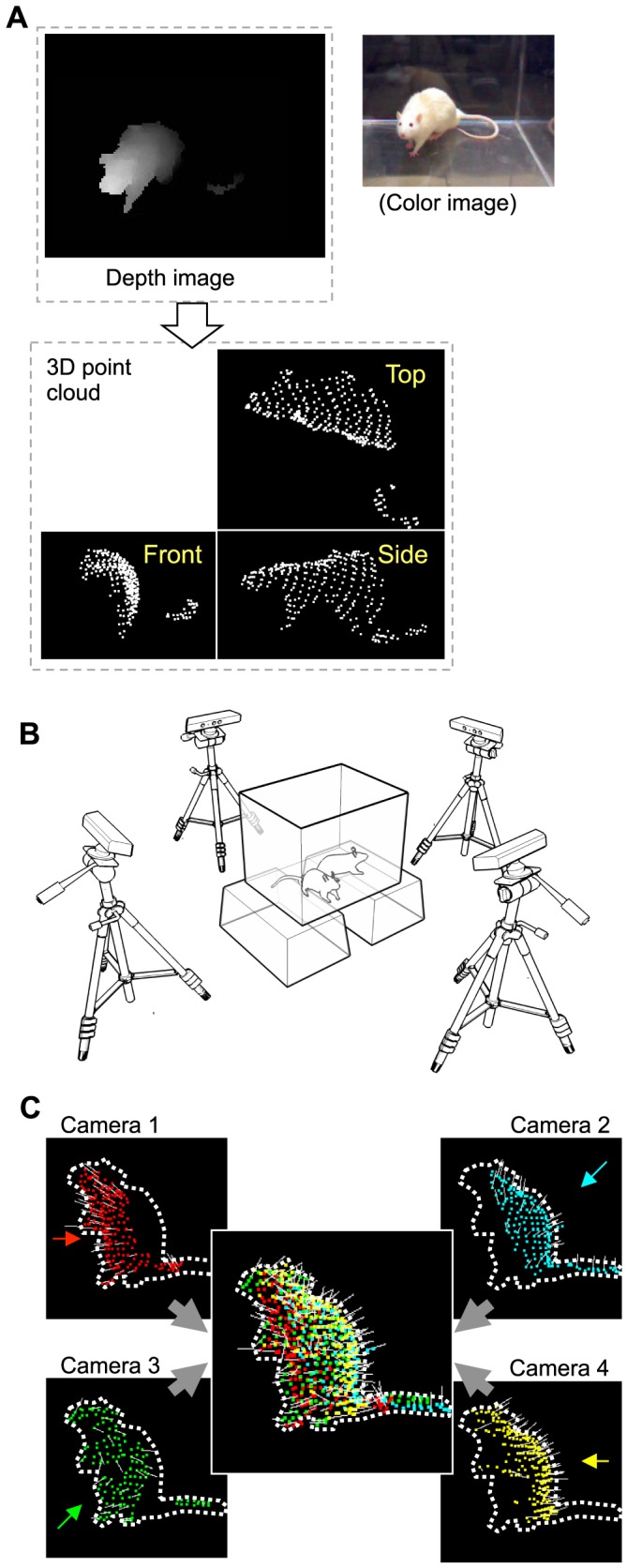
The 3D data acquisition method. A: Examples of a depth image (top left) captured by 1 depth camera, a corresponding color image (top right), and a 3D point cloud extracted from the depth image (bottom). B: The recording setup. Rats in the central recording chamber were captured by 4 depth cameras from different viewpoints. C: An example of a 3D hull of a rat that was reconstructed by merging the point clouds that were captured by the 4 depth cameras. Dotted lines indicate the silhouettes of a captured rat (which is rearing). The surrounding pictures show point clouds that were captured by the 4 depth cameras, with arrows indicating the viewing directions of the cameras; and the central picture shows a merged point cloud. Different point colors represent different cameras used to capture the points and the white line attached to the points represents the surface normal at the points.

To achieve this goal, we first needed to obtain 3D data of the rat behaviors. To obtain these data we used multiple depth cameras, which acted like a 3D scanner; we decided to use depth cameras because they have a fast frame rate (≥30 Hz) and are readily available at a relatively low cost. A depth camera captures a depth image, in which each pixel represents the distance from the camera to the surface of an object in the pixel (Figure 1A, top left). A depth image can then be easily converted into 3D data, whereby a set of points (point cloud) in 3D space represents the surface of an object (Figure 1A, bottom). Because 1 camera cannot cover the entire surface of the objects, we merged point clouds that were captured by 4 depth cameras from different viewpoints to reconstruct the entire surface (Figure 1B, C). Thus, the resultant 3D data were a 3D “hull” of the objects which was represented by a point cloud.

**Figure 2:**
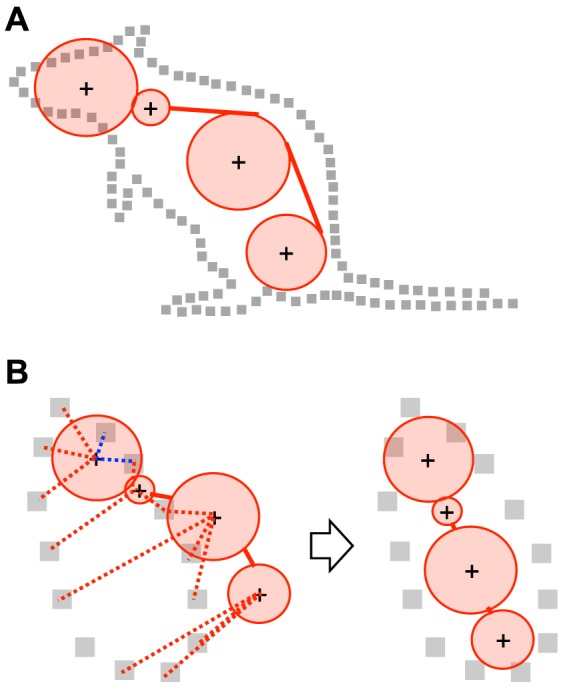
Basic concept of the position estimation algorithm. A. The strategy of the position estimation. To estimate the positions of body parts (shown as cross marks), the skeleton model of a rat (shown in red) is physically fitted into the 3D hull of a rat (shown as gray squares). B: Schematics explaining the physical forces that were assumed to converge the skeleton model into the 3D hull. Left: before converge; right: after converge. Red lines: the attraction forces; blue lines: the repulsive forces.

Once we had acquired the 3D hull of a rat, we then needed to use this to compute the 3D positions of the 4 body parts. Figure 2A shows the basic way in which we achieved this. We developed a 3D model of the rat skeleton using 4 connected spheres, with each sphere corresponding to 1 of the rat's 4 body parts. By physically fitting the model into the 3D hull, the body part positions of the modeled skeleton converged on the actual positions. We implemented this by simulating physical systems in a computer using a physics engine, which is a software library that provides such a physics simulation; thanks to recent developments, fast and easy-to-use physics engines are now freely available. A physics simulation was conducted under the following 3 conditions to converge the skeleton model into the appropriate position inside the 3D hull: 1) there was an attraction force from each body part to each point on the hull, to lead the skeleton model to the 3D hull (Figure 2B, red lines); 2) there was a repulsive force from each point on the hull to each body part of the skeleton model inside the hull, to retain the skeleton model within the 3D hull (Figure 2B, blue lines); and 3) there were some physical constraints (e.g., collision between the skeleton models to prevent them from overlapping), to prevent the skeleton model from taking impossible or unnatural postures. The physics-based fitting algorithm that was used can be considered an extension of the physics-constrained mean shift technique, which was proposed by de Chaumont et al. [10] for analyzing a 2D video of social behavior in mice. Physics-constrained mean-shift is a simple and fast (near real-time) algorithm that can robustly track the positions of the body parts of animals. By repeating the above fitting process for each 3D hull at each point in time, the optimum spatio-temporal pattern of the positions could be determined, allowing various behaviors to be recognized from the pattern.

In the following sections, we explain the details of this 3D video analysis system based on the above concepts in 3 steps: 3D data acquisition, estimation of positions of the body parts in the 3D data, and recognition of behavior from the spatio-temporal pattern of the positions. We then describe 3 experiments that were conducted to validate the use of this system.

### 3D data acquisition

Figure 1B shows the recording setup. A transparent acrylic recording chamber (50×39×39 cm) was placed on transparent footholds (20-cm high), and 4 depth cameras (Kinect for windows, Microsoft, around $200/camera) were placed around the chamber at 3, 6, 9, and 12 o'clock positions. The cameras were connected to a PC (Core i5 750 2.67 GHz processor (4 cores) and 4GB memory). The distance between each camera and the center of the chamber was 60–80 cm and the cameras were 60-cm high. The cameras were tilted at an angle of 15° downward from the horizontal plane. In the following text, a “partial 3D hull” refers to a point cloud that has been converted from a depth image captured by a camera; a “partial 3D hull with normal” refers to a partial 3D hull with estimated surface normal at each point (the surface normal is used to judge whether a body part is inside or outside the hull for the physics-based fitting algorithm); a “full 3D hull” refers to a hull that has been generated by merging the partial 3D hulls with normal from the 4 cameras; and a “3D hull stream” refers to a successive full 3D hull sequence. The camera positions and angles were measured externally to integrate a partial 3D hull of different cameras into a full 3D hull. The camera parameters were further calibrated manually by placing known objects at known positions within the recording chamber.

**Figure 3:**
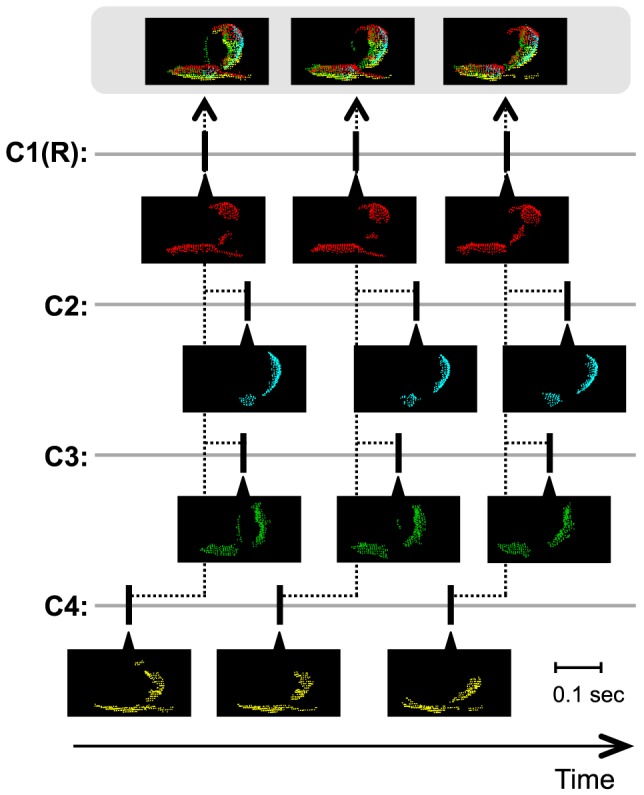
Selection of partial 3D hulls with normal for the reconstruction of a full 3D hull. C: camera, R: reference camera. Example frames with partial 3D hulls constructed from 4 cameras (C1 to C4), and frames with full 3D hulls reconstructions (top) are shown. Thick vertical lines indicate times when the frames were captured. Dotted lines connect selected frames for reconstruction of a full 3D hull. The frames were captured while a male was chasing a female.

Because the cameras were not synchronized and the reconstruction of a full 3D hull from partial 3D hulls takes a little computation time, partial 3D hulls were individually acquired from each camera and stored on the hard disk drive; the 3D hull stream was then reconstructed offline from the stored partial 3D hulls. During the online processing, a depth image (320×240 pixels) from each camera was converted into a partial 3D hull using the functions of Microsoft Kinect SDK version 1.5 (http://www.microsoft.com/en-us/kinectforwindows/) and stored with its timestamp. To reduce the file size, the points outside the recording chamber in the partial 3D hull were removed and the partial 3D hull was downsampled into 1 point/cm^3^ using VoxelGrid filter in Point Cloud Library version 1.6.0 (an open-source library for processing point clouds, http://pointclouds.org/) prior to storage. This online processing works at 30 frames/sec (which is equal to the maximum frame rate of the camera) using the PC described above. During the offline processing, the surface normal at each point of each stored partial 3D hull was calculated using Point Cloud to generate a partial 3D hull with normal. To reconstruct full 3D hulls from these asynchronous partial 3D hulls with normal, an arbitrary camera was selected as a reference, following which the partial 3D hulls with normal of 4 cameras whose timestamps were closest to each timestamp of the reference camera were merged into a full 3D hull (Figure 3). The resultant full 3D hulls were aligned according to the timestamps of the reference camera to generate a 3D hull stream. The programs for both the online and offline processing for the 3D hull stream acquisition were built using Microsoft Visual C++ 2010 (Software S1 and S2, respectively, in File S1).

### Estimation of positions of the body parts in the 3D data

**Figure 4:**
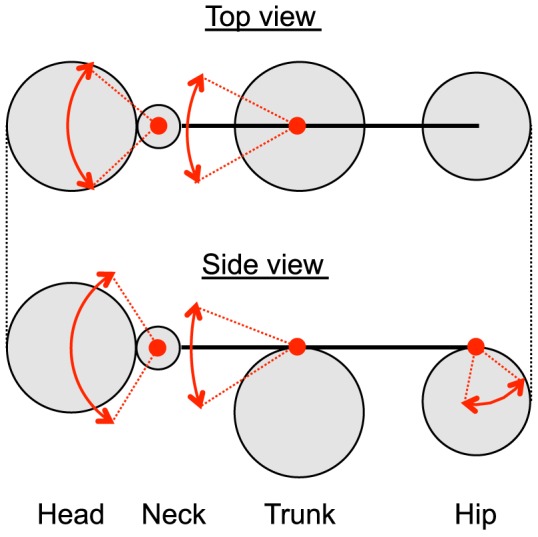
The skeleton model of a rat that was used for position estimation. Red two-way arrows represent the ranges of movement of the joints.

Here, we describe the algorithm that was used to fit 3D skeleton models of rats to a full 3D hull in a frame of 3D hull streams using the physics simulation to estimate the positions of the body parts of rats. The physics simulation was carried out using an open-source physics engine, Bullet Physics Library version 2.8.1 (http://bulletphysics.org/). Figure 4 shows the 3D skeleton model of a rat that was used in the present system. This model consisted of 4 body parts (head, neck, trunk, and hip), which were connected by joints that had certain ranges of movement, as described in Figure 4. Before starting the physics simulation, it was necessary to provide the initial positions of the skeleton models, which needed to be located near to the actual positions for appropriate fitting. These initial positions were set manually for the first frame and were then based on the estimated positions in the previous frames for the following frames. The physics simulation proceeded with small time steps, at the beginning of each of which the velocities of the skeleton models were reset to zero. Attraction forces (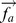) and repulsive forces (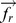) were then calculated and applied as impulses to each body part of each skeleton model.

**Figure 5:**
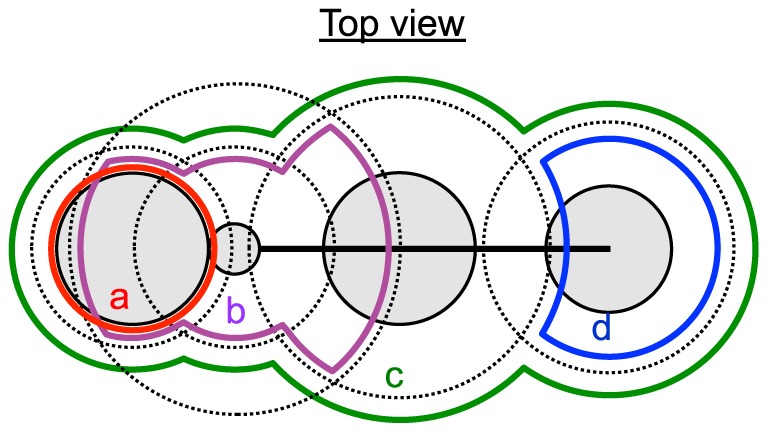
Schematics of regions for selecting points for attraction forces. Each of the regions (a, b, c, and d) is a composition of spherical regions around the centers of the body parts (dotted lines). Each color represents a region.


Attraction forces were applied to body parts from points on the hull, to lead the skeleton model into the hull. However, if all body parts received attraction forces from all points on the hull, every body part would be attracted to the center of gravity of the hull, resulting in the skeleton model taking on an inappropriate posture. Therefore, the head, neck, trunk, and hip received attraction forces only to points within the regions a, b, c, and d shown in Figure 5, respectively. In this way, the head and hip were attracted to the edge of the hull of a rat, the trunk was attracted to the center of the hull of a rat, and the neck was attracted the center between the head and the trunk. The definition of these regions was as follows:

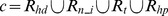
, 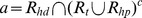



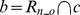
, 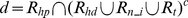 where *R_hd_*, *R_t_*, and *R_hp_* represent spherical regions with certain radiuses around the head, trunk, and hip, respectively; and *R_n_i_* and *R_n_o_* represent spherical regions with certain radiuses around the neck, with a shorter radius for *R_n_i_* than *R_n_o_*. Furthermore, to prevent the skeleton model from being attracted to the hull of the other rat, points that occurred in region c of the other skeleton model were not included in the calculation of attraction forces. Note that the regions shift following the skeleton models during simulation steps, which means the point assignments to the regions (and to the models) changes dynamically. The value and direction of the attraction force for each rat body part (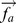) was calculated using the following equation that was applied to the center of the body part:
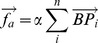
where α is a constant, *B* is the center position of the body part, and *P_i_*(*i* = 1, …,*n*) is a subset of all points in the full 3D hull that fulfill the criteria described above.

Repulsive forces were applied from points on the hull to keep the skeleton model within the hull. However, if the repulsive forces were applied to body parts outside the hull, the skeleton model could not then enter the hull. Therefore, it was necessary to apply the repulsive forces only to the body parts inside the hull. The center of a body part (*B*) was judged to be inside with respect to a point (*Q*) on the hull if the following equation was fulfilled:
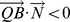
where 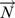 is a surface normal at the point. Furthermore, the repulsive forces were applied only when the body part contacted with the point (hull), i.e., when the following equation was fulfilled:
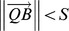
where *S* is a constant that is lower than the radius of the body part. The value and direction of the repulsive force for each rat body part (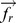) was calculated using the following equation and was applied the center of the body part:
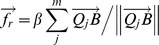
where *β* is a constant and *Q_j_*(*j* = 1, …,*m*) is a subset of all points in the full 3D hull that fulfill the criteria described above. Note that all points were used for calculating the repulsive forces for all body parts for all skeleton models, as opposed to calculating the attraction forces.

In addition to these attraction and repulsive forces, the following 4 physical constraints were assumed to prevent the skeleton model from taking on impossible or less likely postures: I) collision between the skeleton models, II) collision between a skeleton model and the floor of the chamber, III) prevention of rotation of the trunk of a skeleton model along the rostral-caudal axis to ensure that the back was always toward the ceiling, and IV) prevention of the hip from going more anterior than the trunk. Constraints I and II were implemented by using standard functions of Bullet Physics Library, while constraint III was implemented by applying negative feedback force against the rotation and constraint IV was implemented by shifting the horizontal position of the hip to that of the trunk when the hip went more anterior than the trunk.

The above algorithm also can work during close contact between 2 rats. As noted above, points that were contained within regions for 2 rats were ignored from calculations of attraction force (Figure S1, gray squares). Removing the points shared between rats slightly increases the error of estimation for calculating the attraction force. However, because of the other physical constraints that stabilize the model (especially the repulsive force from the points and the collision between the models), the system usually can trace the body parts without large errors.

**Figure 6:**
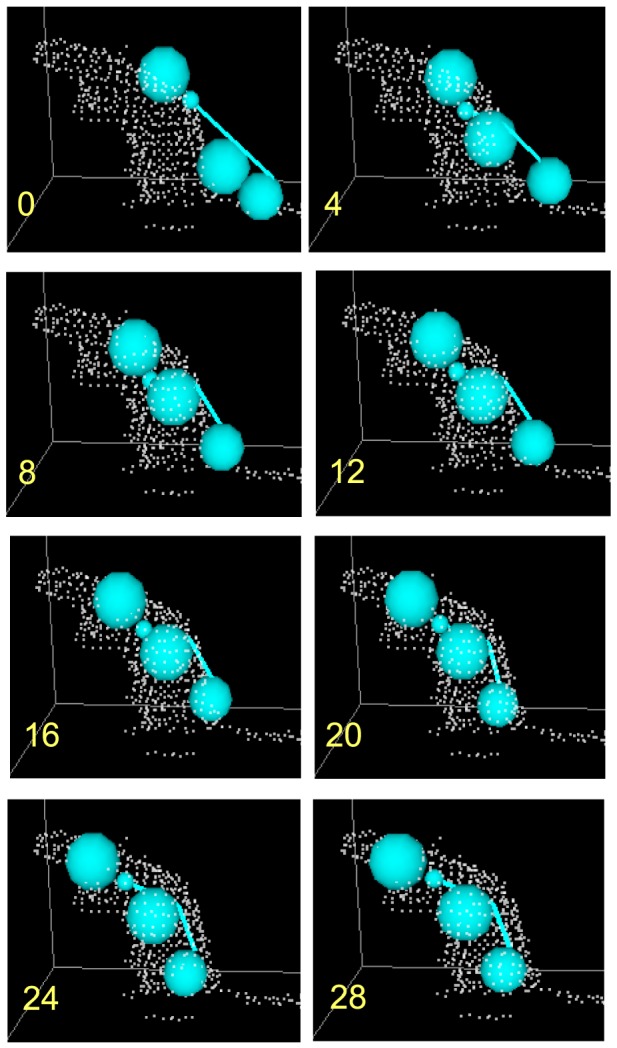
An example of the performance of the fitting algorithm. The 3D hull of the rat is shown as white points (the rat is rearing) and the skeleton model is shown as light blue. A yellow number in each picture represents the corresponding step in the simulation, i.e., the position of the skeleton model in the top left picture corresponds to the initial position, while the position of the skeleton model in the bottom right picture corresponds to the result of the fitting.

The simulation steps were repeated until the simulated physical system reached a steady state in which all body parts shifts in the skeleton models for each step fell below a small value. Figure 6 provides an example of the performance of this fitting algorithm from the start to the steady state. The program for the position estimation was built using Microsoft Visual C++ 2010 (Software S3 in File S1).

### Recognition of behavior based on the estimation

Once the position of the rats had been estimated (see above), various behaviors were recognized from the spatio-temporal patterns of the positions of the centers of the 4 body parts. First, the traces of the centers of the 4 body parts were filtered with a loess filter (time window: 0.5 sec) using the “smooth()” function of Matlab version R2011b (see Figure S2 for examples of smoothed traces). The filtered traces were then used to calculate the velocities of the rats (the velocity of the trunk center along the vector from the hip center to the head center on the horizontal plane), vertical angles of the rats (the angle between the floor and the vector from the hip center to the head center), and horizontal directions of the rats (the direction of the vector from the hip center to the head center on the horizontal plane) in each frame. The filtered trace and these parameters were then used to recognize behaviors in each frame.

**Figure 7:**
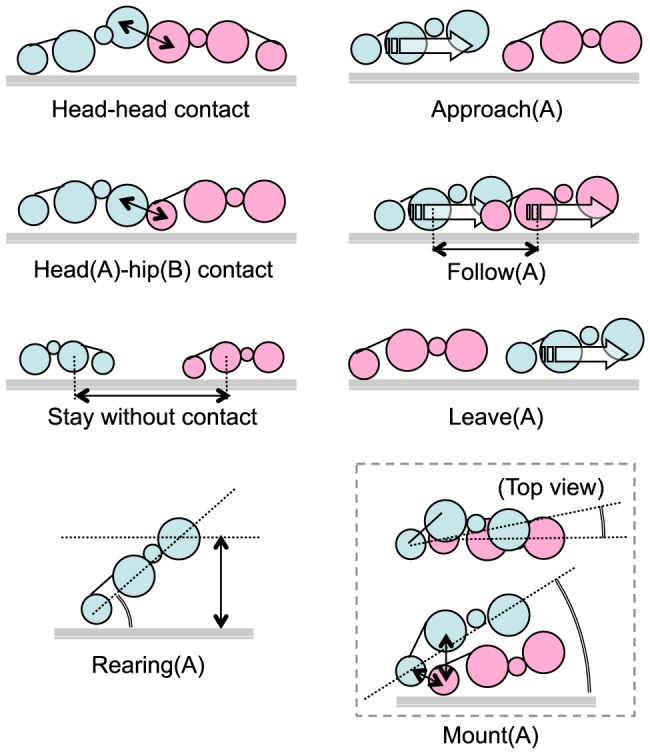
Definition of the behavioral events. Gray line indicates the floor. The light blue model represents Rat A and the pink model represents Rat B. See text for details.

In this study, the following 13 behavioral events between 2 rats (referred to hereafter as “Rat A” and “Rat B”) were defined and recognized (see Figure 7 for illustrations of these behaviors):

*Event 1, Rearing (A)*: height of the head center >14 cm and vertical angle of Rat A>45°.

*Event 2, Head–head contact*: distance between the heads of the 2 rats <8 cm and velocities of the rats <5 cm/s.

*Event 3, Head(A)–hip(B) contact*: distance between the head center of Rat A and the hip center of Rat B <8 cm, and velocities of the rats <5 cm/s.

*Event 4, Stop without contact*: velocities of the rats <5 cm/s and the distance between every body part of the 2 rats >10 cm.

*Event 5, Approach(A)*: velocity of Rat A >10 cm/s, velocity of Rat A > Rat B, and distance between the trunk center of the rats decreases faster than 10 cm/s.

*Event 6, Leave(A)*: velocity of Rat A >10 cm/s, velocity of Rat A > Rat B, and distance between the trunk center of the rats increases faster than 10 cm/s.

*Event 7, Follow*: velocity of the 2 rats >10 cm/s, distance between the trunk center of the rats <20 cm, and difference between horizontal directions of the rats <90°.

*Event 8, Mount(A)*: distance between the hip centers of the 2 rats <10 cm, distance between the trunk center of Rat A and the hip center of Rat B <10 cm, vertical angle of Rat A >20°, and difference between the horizontal directions of the rats <45°. (Note that for the purposes of this system, a mount does not necessarily involve thrusting, as this could not be detected due to the low resolution of the cameras; also mounts with and without intromission and ejaculation were not distinguished.)

*Events 9–13 (Rearing(B)*, *Head(B)–hip(A) contact*, *Approach(B)*, *Leave(B)*, and *Mount(B)*, respectively) were also defined.

After recognizing all the behavioral events in each frame, the event markers were filtered according to the following rules: if the same type of event occurred within an interval of less than 0.3 sec (or 0.5 sec for a mount), the event was considered to have continued during the interval; and if the duration of an event was less than 0.1 sec, the occurrence of the event was ignored. The scripts for the behavioral recognition were made using Matlab version R2011b.

### Experiment 1: Validation of the 3D system using male–male social interactions

To validate the 3D system, the behavior of rats during male–male social interactions was analyzed and compared with data derived from visual observation by the experimenters. For this experiment, 6 adult male Wistar rats weighing 340–390 g (SLC) were used. Three rats were housed per cage at a temperature of 23±1°C and a 12 h light:dark cycle (light on at 7:00 A.M.), and food and water were available *ad libitum*. The behavioral recordings were made between 8:00 P.M. and 9:00 P.M., during the dark phase. Two rats that originated from different cages were placed in the recording chamber and their behavior was recorded for 10 min. After the recording, a 3D hull stream of each trial was reconstructed offline, which was then used to estimate the positions of the body parts. Default program parameters (Tables S1–S2) were used to estimate the positions, with the exception of scaling the sizes of skeleton models, which was dependent on rat size (see Table S3 for actual values used for scaling). These parameters were examined by visually inspecting the fitting results in selected data samples.

Some errors that occurred during model fitting in a single frame continued in subsequent frames and could persist over long durations. These errors were difficult to correct without manual intervention, and were considered a “significant problem.” A significant problem was identified by at least 1 of the following 2 criteria: 1) the skeleton model direction was reversed from the actual direction of the rat 3D hull, or 2) the skeleton models were swapped between the 3D hulls of 2 rats. The frequency of significant problems, however, should be low to save time and the work required for the position estimation process. Therefore, to validate the present system, we first manually determined the frequency of significant problems. We divided each 10 min 3D hull stream into consecutive 1-min periods and processed each period until a significant problem was identified, or until the end of the period. These tests allowed us to calculate the cumulative frequency of significant problems over time. Next, to assess the accuracy of the estimated positions for the skeleton model body parts, we calculated the averaged error of the estimated positions by comparing the skeleton model positions estimated by our system with those estimated by 2 blind experimenters. Nine periods were randomly selected from the 1-min periods that were processed without identifying significant problems using the present system. The estimation errors of the centers of the 4 body parts were calculated using 7 different frames at 0, 10, 20, 30, 40, 50, and 60 sec from the beginning of each period. To test whether the error increased over time (i.e., whether the position of the body parts “drift” from their correct positions), we compared the averaged error among the 7 frames using a one-way repeated measured ANOVA (N = 18). Bonferroni's post hoc test was used to compare between the errors in the first frame and each of the following frames.

To validate the advantages of the present system, we also compared the errors of the present system with the errors of linear interpolation using adjacent frames (e.g., for calculating the position in the frame at 20 sec, the experimenter-estimated positions in the frames at 10 and 30 sec were used). Finally, to determine the computational cost of the position estimation algorithm, we divided the 3D hull streams into consecutive 20-sec periods, and then averaged the computation time required for these 20-sec periods that were calculated on a PC equipped with a Core i5 750 2.67 GHz processor (4 cores) and 4 GB of random-access memory. Periods in which significant problems were detected (see above) were excluded from this analysis.

### Experiment 2: Validation of the 3D system using male–female sexual interactions

The behavior of rats during male–female sexual interactions was analyzed and compared with data obtained through visual observation by the experimenters. For this experiment, an adult sexually experienced male Wistar rat weighing 370–390 g (SLC) and an adult female Wister rat weighing 220–230 g (SLC) were used. The rats were housed in the same conditions as for experiment 1, except that 2 rats of the same sex were housed per cage. The female rat was ovariectomized under intraperitoneal sodium pentobarbital anesthesia (40 mg/kg) more than 1 month before the experiment. The female rats were subcutaneously injected with estradiol benzoate (5 µg/rat) and progesterone (500 µg/rat) 48 h and 4–7 h prior to recording, respectively. The behavioral recordings were made between 8:00 P.M. and 9:00 P.M., during the dark phase, on 2 days at an interval of 22 days. Two and three sexual interaction trials were recorded on days 1 and 2, respectively. During each trial, the male and female rats were placed in the recording chamber and recorded until 1 min after ejaculation. The interval between recordings in a day was >10 min.

After a recording, a 3D hull stream was reconstructed offline for each trial that was used to estimate the body part positions and to identify the behavioral events. Next, the frequency of significant problems per period, the precision of the estimated positions, and the computation time per period were determined using the same procedures described for Experiment 1, except for the period selection process. To calculate the frequency of significant problems, we analyzed the data recorded around the times that rats showed mounting behaviors. The averaged errors were calculated using the frames at −2, −1, 0, +1, and +2 sec from the onset of mounting, and the frame occurring 2 sec before the next mounting. The computation time was estimated using the period between −2 to 2 sec from the onset of mounting. Finally, the positions of the body parts of the male and female were estimated throughout the recording using the 3D system, with some manual interventions. In the manual interventions, significant problems and other large misalignment of body parts (e.g., a body part was located outside the full 3D hull) were fixed. Then, male mounting behavior was recognized based on the spatio-temporal patterns of the positions, as described above. The timing of mounts estimated by the system was compared with those reported by a blind experimenter based on visual observation (in this case, “mount” included all mounts during which there was pelvic thrusting, regardless of whether penile intromission or ejaculation occurred). In addition, the quality of the classifications for physical contacts or movements were tested by examining Event 2 (head-head contact) and Event 4 (approach of a male), respectively. Ten periods were extracted from 5 sexual interaction trials by dividing each trial into halves. The frequency and duration of the 2 events in each period then were counted by the present system and the 2 blind experimenters based on visual observation. Finally, we calculated the correlations for each parameter between the system and each experimenter.

### Experiment 3: Analyzing the effects of AM-251 on male sexual behavior using the 3D system

To test the effectiveness of the 3D system, we used it to analyze the effects of AM-251 (a cannabinoid CB1 receptor antagonist) on male sexual behavior. In this experiment, the sexual behavior of male rats was examined following the administration of either AM-251 (5 mg/kg) or its vehicle (see below). Eighteen adult male Wistar rats weighing 320–360 g (SLC) and 18 adult female Wistar rats weighing 190–200 g (SLC) were used. Rats were housed in the same conditions as those described for Experiment 2, except that 3 rats of the same sex were housed in a single cage. The female rats were used to stimulate male sexual behavior and had been ovariectomized under intraperitoneal sodium pentobarbital anesthesia (40 mg/kg) more than 1 month prior to the experiment; they were then subcutaneously injected with estradiol benzoate (5 µg/rat) and progesterone (500 µg/rat) 48 h and 4–7 h prior to the experiment, respectively. Following training, screening and test sessions were conducted between 8:00 P.M. and 12:00 A.M.

Before testing the effects of the drug, each male rat was sexually trained and screened in 3 successive training sessions and 1 screening session, which occurred at intervals of >4 days. During each training session, a male rat was allowed to freely interact with an estrous female for 60–80 min in a transparent acrylic chamber that was the same size as the recording chamber. During the screening session, the sexual proficiency of each male rat was evaluated in the same environment and using the same procedure as during the testing session. Specifically, a male rat was habituated in the recording chamber for 5 min, following which a female rat was placed in the chamber; their behavior was then observed until the first intromission after the first ejaculation of the male rat. A male rat passed the screening and was used in the following test sessions if it ejaculated within 10 min of being presented with the female and re-initiated copulation (showed intromission) within 10 min of the first ejaculation.

Male rats that passed the screening were randomly assigned to either the AM-251 or the vehicle group. A successive test session was conducted for each male rat. Before the test session, AM-251 and the vehicle were injected in AM-251 and vehicle group rats, respectively. The vehicle consisted of a 1∶2∶7 ratio of Tween 80:dimethyl sulfoxide: 0.9% saline; AM-251 was dissolved in the vehicle at a concentration of 5 mg/mL. Both the vehicle and AM-251 were administrated at a concentration of 1 mL/kg i.p. 30 min before the test session. During each test session, a male rat was habituated in the recording chamber for 5 min, following which a female rat was placed in the chamber; their behavior was then observed until the first intromission after the first ejaculation. The behaviors of the test sessions were recorded by the 3D system, and a 3D hull stream of each trial was then reconstructed offline.

To analyze the data, we first scored the following sexual behavioral parameters (following previous studies [2], [8]) based on visual inspection of the recorded 3D hull stream: frequency and latency of mounts with pelvic thrusting before ejaculation (note that in this instance “mount” only refers to the mount without penile intromission); frequency and latency of penile intromissions before ejaculation; ejaculation latency (i.e., the time from the first intromission to the first ejaculation); and the post-ejaculatory interval (i.e., the time from ejaculation to the next intromission). These parameters were compared between rats exposed to AM-251 and the vehicle using a paired t-test.

**Figure 8:**
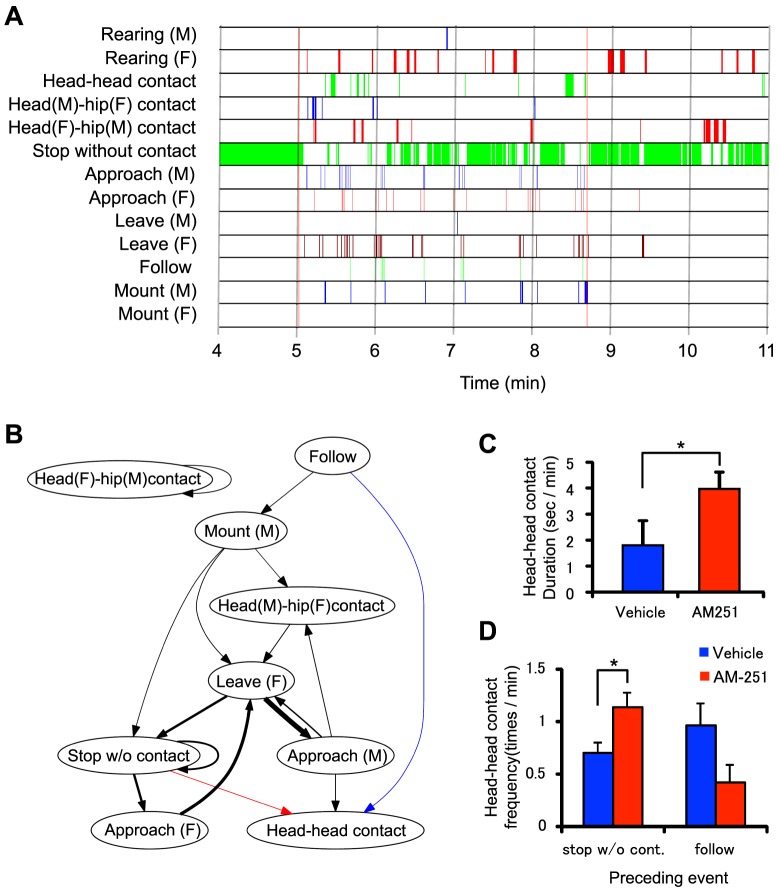
Examples of analysis of behavioral events. A: An example of a chronogram of the occurrence of behavioral events (a test session of AM-251). In the chronogram, each line represents the time at which each event occurred. M: male; F: female. The first red vertical line indicates the time at which the female was introduced, while the second red vertical line indicates the timing of ejaculation. B: Mean transitional behavioral graphs for all of the test sessions. The colored edges (arrows) indicate significant differences in probability of transitions between the 2 groups (unpaired t-test, p<0.05); blue and red arrows indicate that probability was higher in the vehicle AM-251 groups, respectively. Black arrows indicate that probability was not significantly different between the 2 groups. The thickness of the edge (arrow) is proportional to the corresponding probability of transition. Only the edges with a probability of transition >3% are shown. C: Comparison between the 2 groups for durations of head-head contact events during copulatory period, * p<0.05. D: Comparison between the 2 groups for frequency of transition from stop without contact to head-head contact (left), and the transition from follow to head-head contact (right),*p<0.05.

The positions of the body parts in the 3D hull streams were then estimated and the behavioral events were recognized using these positions by the 3D system, in the way described above. The position estimation process for 1 session usually took 1.5 times its duration. About 5–10 manual interventions were performed during the process. These 3D traces of the centers of the body parts and behavioral event markers were used to analyze the data, which were separated into 3 periods: solitary period (2 min before introduction of the female), copulatory period (time between the first mount after the female introduction and the first ejaculation), and post-ejaculatory period (2 min after the first ejaculation). To display the time of occurrence of the events throughout each of the test sessions, we constructed a chronogram of the events (Figure 8A). A paired t-test was then used to compare the frequency and duration of the behavioral events during the interaction period in rats injected with AM-251 and the vehicle, to determine whether the administration of AM-251 altered the occurrence of the behavioral events during sexual interactions.

To check whether the rats' behavioral choices were changed by AM-251 transitional behavioral graphs were drawn according to a method of de Chaumont et al. [7], (Figure 8B). In these graphs, each node represents a behavioral event and the weight of each edge represents the probability of transition from the event on the origin of the edge to the event on the target of the edge. The probability of the transition from Event *i* to Event *j* (*P_ij_*) was computed as follows:
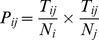
where *N_i_* and *N_j_* represent the number of occurrences of Events *i* and *j*, respectively, and *T_ij_* represents the number of transitions from Event *i* to *j*. A transition from Event *i* to *j* was counted if Event *i* was followed by Event *j*, there were no other events between the transition, and the interval between the events was <1 sec. All probabilities of the possible transitions for rats that had been injected with AM-251 and the vehicle were compared using an unpaired t-test.

The present system enabled us to analyze 3D traces of body parts during sexual behavior. Using this advantage, we examined 2 aspects of male rat behavioral activity level. First, we measured path length as an index of movement (locomotion). Second, we measured the averaged trunk height when male rats remained stationary (velocity <5 cm/sec) as an index of their readiness to move. The trunk height is typically high when the rat is assuming a posture in preparation for running, but is typically low at rest. The path length and the averaged trunk height was computed in each of the 3 periods (the solitary, copulatory, and post-ejaculatory periods), and were compared between the 2 treatment groups using a 2-way ANOVA with 6 conditions: the 3 periods ×2 drugs (AM-251: N = 9; vehicle: N = 8). To eliminate potential height changes resulting from rearing, grooming, or mounting, periods in which mounting was recognized, or when the vertical angle of the rat was >25°, were excluded from computations of the average trunk height.

### Ethics statement

All rats used in this study were treated in strict compliance with the United States Public Health Service Policy on Human Care and Use of Laboratory Animals, National Institutes of Health Guide for the Care and Use of Laboratory Animals, and Guidelines for the Care and Use of Laboratory Animals at the University of Toyama. All experimental procedures were approved by our institutional committee for experimental animal ethics (the ethics committee for animal experiments in University of Toyama) (Approval number: A2012MED-16). All surgery was performed under sodium pentobarbital anesthesia, and all efforts were made to minimize suffering.

## Results

### Experiment 1: Validation using male–male social interactions

**Figure 9:**
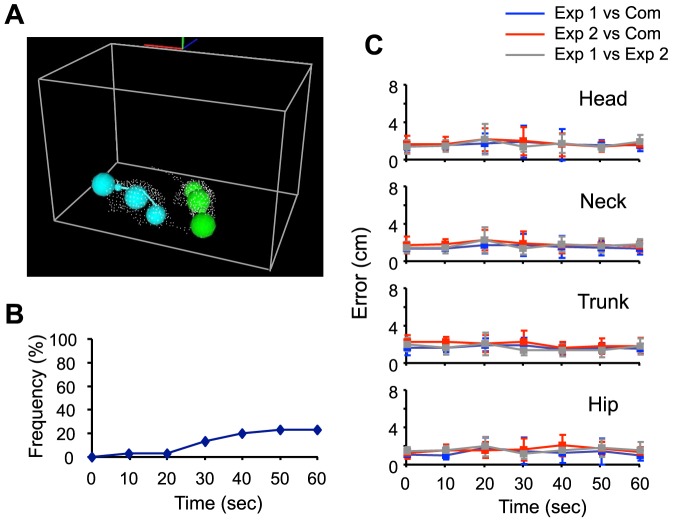
Validation of position estimation during social interaction. A: An example of the estimated positions of body parts is shown, where the spheres, points and gray wireframe cube represent the models, the reconstructed 3D image, and the recording chamber, respectively. The corresponding movie is available as Movie S1. B: Cumulative frequency of occurrence of significant problems over 60 sec. C: Averaged errors of estimated positions for the head, neck trunk, and hip at each timing in the 1-min period. Exp 1: the experimenter 1; Exp 2: the experimenter 2; Com: the present system. Error bars: SD.

Figure 9 provides an example of the estimated positions of the body parts of 2 male rats during a social interaction (see also Movie S1 for an example of the estimated positions throughout a 20-sec period). The average computation time for each 20-sec period was 21.86±1.40 sec (mean ± SD). Figure 9B shows the cumulative frequency of significant problems over 1-min periods. Significant problems were not observed in more than 75% of the periods. Figure 9C shows the differences in the center positions of the 4 body parts between the 3D system and the experimenters. The results indicated the 3D system showed errors around 2 cm as compared with the experimenters. However, given that the resolution of the 3D hull was 1 point/cm3, the length of each rat's body was around 20 cm, and the error between the 2 experimenters was around 2 cm, this is not particularly high. We did not find significant differences in errors among the 7 frames over 60 sec (one-way repeated measures ANOVA, p>0.05), suggesting the error did not accumulate over time. Furthermore, the errors between the position estimated by the experimenters and those estimated by linear interpolation were around 10 cm (Figure S3A), and significantly larger than the errors of the present system in all frames (paired t-test, p<0.01), thus demonstrating an advantage for the present algorithm. Therefore, these results indicate the 3D system can precisely estimate the body part positions of 2 male rats during social interactions, almost in real-time, with very few manual interventions.

### Experiment 2: Validation using male–female sexual interactions

**Figure 10:**
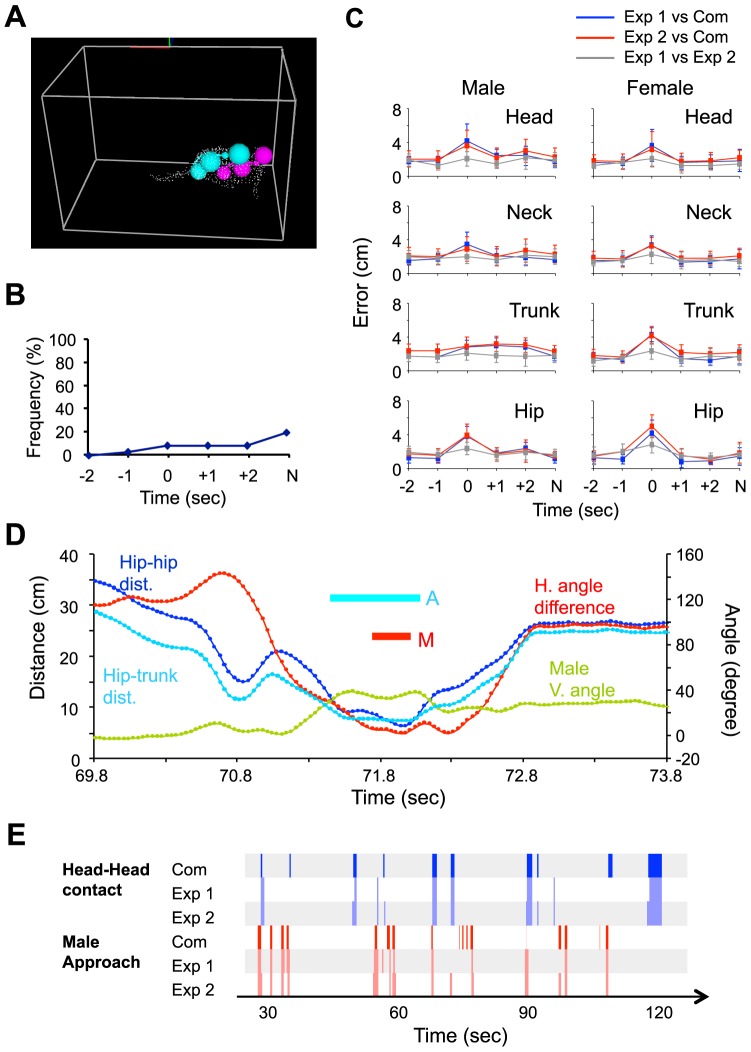
Validation of position estimation during sexual behavior. A: An example of the estimated positions of body parts during sexual behavior. The corresponding movie is available as Movie S2. B: Cumulative frequency of occurrence of significant problems around mounting. Horizontal axis indicates the time from the onset of mounting. N: −2 sec from onset of the next mounting. C: The averaged errors of estimated positions for the head, neck, trunk, and hip of male and females around mounting. Exp 1: the experimenter 1; Exp 2: the experimenter 2; Com: the present system. Error bars: SD. D: An example of the time-course of parameters used for detecting mounting around an occurrence of a mount. Hip–hip dist.: distance between the hips; hip–trunk dist.: distance between the female hip and the male trunk; H. angle difference: angular difference between the horizontal direction of rats; male V. angle: vertical angle of the male rat; A: time period when the male rat mounted, as automatically detected by the 3D system; M: time period when the male rat mounted, as visually observed by the experimenter. E: An example chronogram for the head-head contact event (blue) and the male approach event (red) marked by the present system (Com) and 2 blind experimenters (Exp 1 and 2).

Figure 10A provides an example of the estimated positions of the body parts of a male and female rat during a sexual interaction (see also Movie S2 for an example of the estimated positions throughout a 4-sec period around a copulatory behavior). The average computation time for each 4-sec period around a copulatory behavior was 4.84±0.26 sec (mean ± SD). Figure 10B shows the cumulative frequency of significant problems over the period between −2 sec from the mount to −2 sec from the next mount. Significant problems were not observed in more than 80% of the periods. Figure 10C shows the differences in the center positions of the 4 body parts between the 3D system and the experimenters. The results indicate the 3D system had an error of around 2–4 cm (error is highest at the onset of mounting). However, given that the resolution of the 3D hull was 1 point/cm3, the length of each rat's body was around 20 cm, and the error between the 2 experimenters was around 2 cm, this also was not particularly high. In most cases, there were significant differences in errors among the 7 frames sampled around mounting (one-way repeated measured ANOVA, p<0.05). However, there was no significant difference in errors between the initial (−2 sec) and last (N) frames, suggesting that the error did not accumulate over time (Bonferroni's test, p>0.05). Although the error at the onset of mounting was relatively high (around 4 cm), the values were still much lower than the errors of linear interpolation (Figure S3B and C; around 15 cm). The errors of the linear interpolation algorithm were significantly larger than those for the present system in every frame (paired t-test, p<0.01), demonstrating the advantage of the present algorithm. These results indicate that, although sexual interactions involve close contact between rats (i.e., mounting), the 3D system is able to accurately process these interactions.

By using the trace of the positions throughout the recording period as estimated by the 3D system (see Movie S3, which presents a demonstration of how the position estimation process was performed throughout the entire recording period), the timing of the occurrence of mounting was checked (based on the definition shown in Figure 7). Figure 10D shows an example of the time-course of parameters that was used to detect the mounting event around the occurrence of a mount. Among 42 onsets of mounting that were detected by the system, 41 were close to those judged by the experimenter (with a difference of <1 sec). In the frames in which the remaining 1 mounting event was detected, the rats were in a similar posture as during mounting but no pelvic thrusting was observed. The average errors for the onset, offset, and duration of mounting were −0.24±0.16, 0.14±0.22, and 0.38±0.30 sec (mean ± SD), respectively. These results indicate that mounting can be automatically detected using the positions of the body parts of rats estimated by the 3D system, although the system cannot discriminate between mounts with and without thrusts. We also compared the head-head contact and male approach events detected by the present system with those marked by the experimenters. Figure 10E shows an example of a chronogram of these event markers. More than half of the event makers detected by the present system overlapped with those marked by experimenters. The frequency and duration of the events estimated by the present system were highly correlated with those estimated by the experimenters (r2>0.65, p<0.01; see Figure S4 in detail for the results).

### Experiment 3: Effects of AM-251 on male sexual behavior

Table 1 shows differences in the sexual behavioral parameters between rats injected with AM-251 and the vehicle, as measured by visual observation. A total of 17 of the 18 male rats passed the screening and were included in the test session. Of these 17 rats, 8 and 9 were assigned to the vehicle and AM-251 groups, respectively. The number of intromissions for the AM-251 group tended to be smaller than those for the vehicle group (unpaired t-test, p = 0.052). The post ejaculatory interval was significantly longer in the AM-251 group than in the vehicle group (unpaired t-test, p = 0.012).

**Table 1:** Comparison of sexual behavioral parameters of the male rats injected with the vehicle and AM-251, based on visual inspection.

	Vehicle	AM-251
Number of mounts	2.00±0.46	3.11±0.79
Number of intromissions	7.75±0.98	5.52±0.72^+^
Mount latency (sec)	14.88±7.35	54.04±31.71
Intromission latency (sec)	22.55±9.31	76.85±31.90
Ejaculation latency (sec)	183.70±27.37	235.29±64.65
Post-ejaculatory interval (sec)	254.33±6.92	308.75±16.85*

Data are presented as mean ± SEM. ^+^, * tendency and significant difference from the vehicle condition (p<0.10 and p<0.05, respectively).

Figure 8A shows an example chronogram of the events estimated by the 3D system during a test session for 1 rat in the AM-251 group. The rat displayed repetitive pursuits and mounting during the copulatory period. The statistical comparison revealed that the duration of head-head contact was significantly longer in the AM-251 group than the vehicle group (unpaired t-test, p = 0.028; Figure 8C). Figure 8B indicates the mean transitional behavioral graphs for all rats. The colored arrows indicate significant differences between the 2 groups. The blue arrow indicates that probability of transition from follow to head-head contact was significantly smaller in the AM-251 group (unpaired t-test, p = 0.024), whereas the red arrow indicates the probability of the transition from stop without contact to head-head contact was significantly higher in the AM-251 group (unpaired t-test, p = 0.0037). The black edges indicate that transition was observed in both groups. These results again suggest that the drug affected head-head contact events. Furthermore, the frequency of the transition from stop without contact was significantly greater in the AM-251 group than in the vehicle group (Figure 8D).

**Figure 11:**
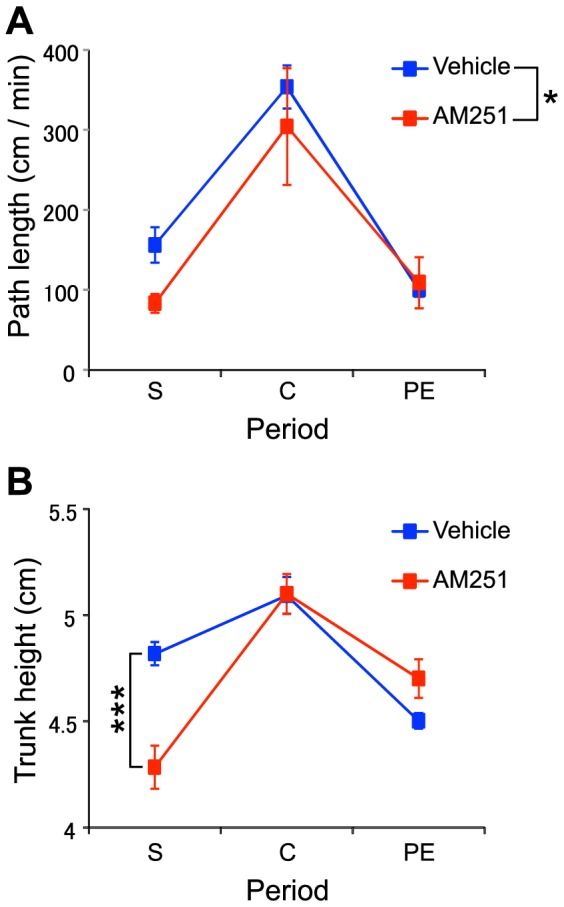
Behavioral activity in Experiment 3 calculated based on the 3D tracking. A: Comparison of the path lengths traveled between the test periods and treatment groups. B: Comparison of the averaged trunk height between the test periods and treatment groups, *** p<0.001, Error bars: SEM; S: solitary period; C: copulatory period; PE: post-ejaculatory period, *p<0.05.

Behavioral activity estimated by the 3D body part measurements also revealed significant differences between the 2 groups. Figure 11A shows the path length in each period for each group. Statistical analysis by 2-way ANOVA indicated that there were significant main effects of period [F (2, 30) = 63.79, p = 1.57×10−11] and drug [F (1, 15) = 5.28, p = 0.036]. Post-hoc multiple comparisons (Ryan's method) revealed that path length was significantly longer during the copulation period than in the other periods, and that the path length was significantly longer in the vehicle group. Figure 11B shows the averaged trunk height when the rat did not move in each period for each group. Statistical analysis by 2-way ANOVA indicated that there was a significant main effect of period [F (2, 30) = 26.76, p = 2.14×10−7], and a significant interaction between period and drug [F (2, 30) = 10.66, p = 3.19×10−4]. Post-hoc multiple comparisons (Ryan's method) revealed that the averaged trunk height was significantly higher during the copulation period than in the other periods. Post-hoc tests by simple main effect revealed the average trunk height was significantly lower in the AM-251 group during the solitary period (p = 1.29×10−4).

**Figure 12:**
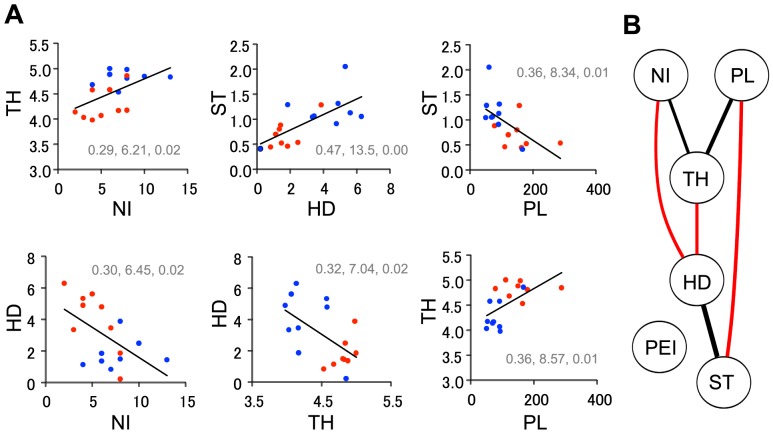
Correlations between the parameters measured in Experiment 3. A: Relationships between the pairs of parameters with significant correlations. Blue and red points indicate data from rats in the vehicle and the AM-251 groups, respectively. NI: number of intromission; HD: the duration of head-head contact (sec/min); ST: the frequency of the transition from stop without contact to head-head contact (/min); PL: the path length during the solitary period (cm/min); TH: the averaged trunk height during the solitary period (cm). The inset numbers represents the results of simple regression analysis; Left: r^2^ value; Middle: F value [F(1,15)]; Right: p-value. B: Summary of the correlation analysis. The curved lines in the graph represent the significant correlations between parameters. The thickness of a curved line is proportional to the r^2^ value, whereas the colors indicate positive (black) and negative (red) correlations. PEI: post-ejaculatory interval.

Finally, we examined correlations among the following behavioral parameters affected by AM-251: number of intromissions (NI), post-ejaculatory interval (PEI), the duration of head-head contact (HD), the frequency of the transition from stop without contact to head-head contact (ST), the path length during the solitary period (PL), and the averaged trunk height during the solitary period (TH). Figure 12A illustrates significant correlations among these parameters (simple linear regression analyses, p<0.05). These results indicate that, except for post-ejaculatory period, each parameter correlated with at least one other parameter (Figure 12B).

## Discussion

### Summary of results

The 3D video analysis system developed in this study was able to estimate precisely the 3D positions of the body parts of 2 rats during social and sexual interactions with few manual interventions. Furthermore, the system could compute the traces of non-painted rats during mounting, although this analysis required a few manual interventions. This is the first computerized analyzing system that offers these advantages. We used this system to analyze the effect of AM-251 on the sexual behavior of male rats, the results of which indicate that the simultaneous analysis of many behaviors using the 3D positions of body parts can provide novel insights into the characterization of sexual behavior. Thus, this 3D system will also prove effective in behavioral studies.

### Effect of AM-251 on male sexual behavior

A previous study based on visual observation reported facilitatory effects of AM-251 on sexual behavior by showing a decreased number of intromissions before ejaculation and decreased ejaculation latencies [8]. Although number of intromissions tended to be decreased by AM-251 in the present study (Table 1), ejaculation latency was not altered. Since the mean ejaculation latencies in the control and AM-251 groups were comparable to those for rats injected with AM-251 in a previous study [8], absence of AM-251 facilitatory effects might be ascribed to a ceiling effect in the present study. The endocannabinoid system is thought to be involved in stress-induced suppression of sexual behavior, and its antagonists reverse this stress-induced suppression [9]. Consistent with this idea, AM-251 decreased activity levels, as indicated by movements, path lengths, and trunk heights (Figure 11), thus suggesting that AM-251 might have a relaxing effect on animals. On the other hand, AM-251 also increased the duration of head-head contact (Figure 8C) and altered the probability of transition to head-head contact (Figure 8B, D). This finding suggests that AM-251 might increase affiliated behaviors, which is consistent with a previous study in which cannabinoid agonists decreased social behaviors [11]. However, pharmacological interventions on cannabinoid receptors are sensitive to drug doses and show a biphasic dose-response curve [12]. Further studies are required to clarify the effects of cannabinoid antagonists on sexual behaviors.

In contrast, significant correlations among the different behavioral parameters were observed during sexual behavior. Although the interpretation of these correlations is difficult, the present results at least indicate that one type of motor act or behavioral state is correlated with other motor acts. Given that each goal-directed behavior (e.g., sexual behavior) consists of a sequence of different motor acts, the present results suggest that 3D analysis of animal behaviors could characterize different goal-directed behaviors.

### Comparison with previous methods

Previous studies have also reconstructed 3D images of a rodent using a single depth camera [13] or multiple RGB cameras [14]. However, these previous systems were only used to estimate the position of the center of an animal, rather than the positions of the body parts, and were designed to analyze only 1 animal. Therefore, these systems could not be directly applied to the analysis of interactions between 2 animals.

The 3D analysis system outlined in this study used a position estimation algorithm that could be considered as an extension of the physics-constrained mean-shift, which was proposed by de Chaumont et al. (2009) for the 2D video analysis of social interactions in mice. Since a 3D skeleton model has greater freedom than a 2D skeleton model, we introduced several assumptions in our modeling in addition to the basic strategies proposed by de Chaumont et al. [10], to stabilize the 3D skeleton model: repulsive forces to retain the model inside the body surface (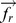), collision between the model and the floor (constraint II), and some constraints on the movement of specific body parts (the constraints III and IV). We also used different types of joints in the model: in de Chaumont et al. 's [10] system, an unnaturally long sliding joint at the neck was assumed to fit the skeleton model to the 2D silhouette, whereas in the present system, only joints with angular movement were used, enabling the reconstruction of more natural movements of an animal.

Human motion capture is an active research area because of its potential wide applications (e.g., surveillance, computer interface, diagnostics of orthopedic patients) [15], [16], [17]. A few systems have been proposed for estimating the poses of closely interacting people without the use of any markers [18], [19]. However, these systems have limitations, such as long computation times [18] or the necessity to use different textures between individuals [18], [19], and so may not be directly applicable to the analysis of interacting rats. However, it is possible that some algorithm proposed for human motion capture may be useful for improving the present system, as discussed in the next section.

### Limitations of the 3D system and future work

From the low-resolution 3D images that were captured by the system described in this study, it was difficult to recognize whether the back of a rat was facing the ceiling or the floor, and so it was assumed that the back was always facing the ceiling (constraint III). Although this assumption would usually be correct, it would be incorrect in some situations, such as during fighting or playing. The present system also requires manual intervention to set the approximate positions of the models in the first frame and when significant problems occur during estimation. Both of these limitations could be improved through the detection of faces, paws, and tails of rats in simultaneously captured color images using a pattern recognition algorithm, and utilizing these in combination with the presently used physics-based position estimation algorithm to estimate the postures and positions of rats. Such a strategy, which has already been proposed for use in human motion capture [20], would also enhance the robustness of tracking, and allow the analysis of fast and complex movements such as aggressive behavior.

The depth cameras in the present system were not synchronized (Kinect does not provide this function by itself). Synchronization would reduce the errors in position estimation in the present system (e.g., if the partial 3D hull at different timing were integrated when a rat is running as shown in Figure 3, the rat body length in the full 3D hull would be slightly longer or shorter than actual one). One possible improvement for the synchronization is to use external triggers set at desired shooting frequency and connected to all the cameras.

Although it would be interesting to apply this 3D system to the behavioral analysis of transgenic mice, this would currently be impossible due to the low resolution and low frame rate of the depth cameras (Kinect can collect depth data between 0.4 to 4 m from the camera with 0.3 cm to 7 cm resolution. The resolution depends on the distance from the camera (longer distance, lower resolution; around 1 cm resolution at 2 m from the sensor). The maximal frame rate is 30 Hz). However, depth cameras with higher resolutions and frame rates are available (e.g., SR4000, MESA Imaging AG; CamBoard nano, PMD Technologies GmbH), although they are more expensive. Therefore, it may be possible to analyze mouse interactions using such a camera. It would also be interesting to modify the present system for application with primates, which show more sophisticated social behaviors.

Automation of the behavioral analysis of interacting animals opens the way for radical new approaches to behavioral studies. First, it will enable us to analyze long-term interactions, e.g., the development of social relationships over several days, although additional data compression would be required for such long-term recording. Second, it will allow real-time feedback of a specific behavior, which, when combined with a technique that controls brain activity at a high temporal resolution [e.g. optogenetics [21]] could help to clarify which neural functions are responsible for the behavior. The position estimation process, which is the most computationally expensive process among the processes in the present system, ran near to real-time speed, and so parallel computation of data acquisition, position estimation, and behavior recognition would allow the system to provide real-time feedback.

## Conclusion

In this study, we introduce a novel system that can estimate the 3D positions of some body parts of rats during social and sexual interactions, and analyze behaviors based on the spatio-temporal patterns of these positions. This was achieved through the use of multiple cameras at different viewpoints and a physics-based fitting algorithm. Further improvements to the system would allow the recording of mice or primates, long-term recording, and real-time feedback on a specific social or sexual behavior, which would open the door to new approaches to investigate the neuroscience of social and sexual behavior.

## Supporting Information

Figure S1 **A schematic indicating the point assignments for a 3D hull to each skeleton model for calculating the attraction force during close contact between rats.** Blue and red lines indicate the region c for the upper (light blue) and lower (pink) models, respectively. The points for the 3D hull are represented by the squares. Light blue and pink colored points are assigned to the upper and lower models, respectively.
(EPS)

Figure S2 **Smoothing with the loess filter.** Traces for the center of the head (A), neck (B), trunk (C), and hip (D) of a male (blue) and female (red) around copulatory behavior (the scene corresponds with Movie S2), before (left) and after (right) smoothing with the loess filter.
(EPS)

Figure S3 **Comparison of errors between the present system and the linear interpolation algorithm.** A: The errors during social interaction (Experiment 1). B: The errors of male body parts during sexual interaction (Experiment 2). C: The errors of female body parts during sexual interaction (Experiment 2).
(EPS)

Figure S4 **Correlations between parameters of behavioral events estimated by the present system and those estimated by two experimenters.** A: The correlations of the frequency (upper) and duration (lower) of the head-head contact event. Com: the present system; Exp 1 and 2: Experimenter 1 and 2; the numbers on top-right the graphs indicate the r^2^ (left) and F values (right; degree of freedom  = 2 and 8). **p<0.01 ***p<0.001. B: The correlations between the frequency (upper) and duration (lower) of the male approach event (terms are the same as in A).
(EPS)

Table S1 The Default parameters of the skeleton model.
(DOC)

Table S2 Parameters for physics-based fitting process.
(DOC)

Table S3 Scaling factor for scaling the skeleton models used in Experiment 1–3.
(DOC)

File S1 The file includes Software S1 (for recording partial 3D hulls), S2 (for converting partial 3D hulls to a 3D hull stream), S3 (for tracking body parts in a 3D hull stream) and S4 (for behavioral recognition based on traces of body parts), manuals for the software and sample data.
(ZIP)

Movie S1 An example of the estimated body parts positions throughout a 20-sec period during social interaction.
(MP4)

Movie S2 An example of the estimated body parts positions throughout a 20-sec period during sexual interaction.
(MP4)

Movie S3 A demonstration of how the position estimation process was performed throughout the entire recording period using software.
(MP4)
