# Supplementary material for: A 3D-Video-Based Computerized Analysis of Social and Sexual Interactions in Rats
Source: PLoS One. 2013 Oct 30;8(10):e78460. doi: 10.1371/journal.pone.0078460 (PMC3813688; doi:10.1371/journal.pone.0078460)
Supplement: Table S1 — The Default parameters of the skeleton model. (DOC) [file pone.0078460.s005.doc]

**Table S1. The Default parameters of the skeleton model.**

| Physical Radius of body parts | Head | 3.0 cm |
| --- | --- | --- |
|  | Neck | 1.0 cm |
|  | Trunk | 3.0 cm |
|  | Hip | 2.5 cm |
| Distance between body parts | Neck - Trunk | 8.0 cm |
|  | Trunk – Hip | 5.0 cm |
| Radius for the repulsive force  (*S* in the text) | Head | 1.0 cm |
|  | Neck | 1.0 cm |
|  | Trunk | 1.7 cm |
|  | Hip | 2.0 cm |
| Radius for the regions for the attraction force | Head (*R_hd_* in the text) | 4.0 cm |
|  | Neck (*Rn_i*, *Rn_o* in the text) | 4.0, 6.5 cm |
|  | Trunk (*R_t_* in the text) | 6.0 cm |
|  | Hip (*R_hp_* in the text) | 5.0 cm |
| Min and Max of joint angle | Neck-Head (left to right) | -45° to 45° |
|  | Neck-Head (dorsal to ventral) | -60° to 60° |
|  | Neck-Trunk (left to right) | -30° to 30° |
|  | Neck-Trunk (dorsal to ventral) | -20° to 30° |
|  | Trunk-Hip (front to back) | -10° to 60° |
