# Supplementary material for: A 3D-Video-Based Computerized Analysis of Social and Sexual Interactions in Rats
Source: PLoS One. 2013 Oct 30;8(10):e78460. doi: 10.1371/journal.pone.0078460 (PMC3813688; doi:10.1371/journal.pone.0078460)
Supplement: Table S2 — Parameters for physics-based fitting process. (DOC) [file pone.0078460.s006.doc]

**Table S2. Parameters for physics-based fitting process.**

| Constant for the attraction force (*α *in the text) | 0.4 Ns/m |
| --- | --- |
| Constant for the repulsive force (*β *in the text) | 0.2 Ns/m |
| Negative feedback force against the rotation (constraint III in the text) | 0.005 Ns |
| Duration of simulation a step of physics-simulation (ΔT) | 0.1 sec |
| Threshold of shift of body parts to judge steady-state | 1 cm/sec |
| Weight of each body parts | 1 kg |
