# Supplementary material for: A 3D-Video-Based Computerized Analysis of Social and Sexual Interactions in Rats
Source: PLoS One. 2013 Oct 30;8(10):e78460. doi: 10.1371/journal.pone.0078460 (PMC3813688; doi:10.1371/journal.pone.0078460)
Supplement: Table S3 — Scaling factor for scaling the skeleton models used in Experiment 1–3. (DOC) [file pone.0078460.s007.doc]

**Table S3. Scaling factor for scaling the skeleton models used in Experiment 1-3.**

| Experiment 1 | All rats: 100% |
| --- | --- |
| Experiment 2 | All males: 100%, All females: 85% |
| Experiment 3 | All males: 95%, All females 85% |

The lengths listed in Table S1 were multiplied with each of these values to scale the model.
