# Supplementary figures and images for: A 3D-Video-Based Computerized Analysis of Social and Sexual Interactions in Rats
Source: PLoS One. 2013 Oct 30;8(10):e78460. doi: 10.1371/journal.pone.0078460 (PMC3813688; doi:10.1371/journal.pone.0078460)

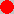

Supplement: File S1 — The file includes Software S1 (for recording partial 3D hulls), S2 (for converting partial 3D hulls to a 3D hull stream), S3 (for tracking body parts in a 3D hull stream) and S4 (for behavioral recognition based on traces of body parts), manuals for the software and sample data. (ZIP) [file pone.0078460.s008.zip › Software S1 - 3DRecorder/source files/3DRecorder/res/bitmap1.bmp]

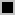

Supplement: File S1 — The file includes Software S1 (for recording partial 3D hulls), S2 (for converting partial 3D hulls to a 3D hull stream), S3 (for tracking body parts in a 3D hull stream) and S4 (for behavioral recognition based on traces of body parts), manuals for the software and sample data. (ZIP) [file pone.0078460.s008.zip › Software S1 - 3DRecorder/source files/3DRecorder/res/bitmap2.bmp]

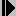

Supplement: File S1 — The file includes Software S1 (for recording partial 3D hulls), S2 (for converting partial 3D hulls to a 3D hull stream), S3 (for tracking body parts in a 3D hull stream) and S4 (for behavioral recognition based on traces of body parts), manuals for the software and sample data. (ZIP) [file pone.0078460.s008.zip › Software S3 - 3DTracker/source files/3DTracker/res/next.bmp]

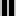

Supplement: File S1 — The file includes Software S1 (for recording partial 3D hulls), S2 (for converting partial 3D hulls to a 3D hull stream), S3 (for tracking body parts in a 3D hull stream) and S4 (for behavioral recognition based on traces of body parts), manuals for the software and sample data. (ZIP) [file pone.0078460.s008.zip › Software S3 - 3DTracker/source files/3DTracker/res/pause.bmp]

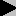

Supplement: File S1 — The file includes Software S1 (for recording partial 3D hulls), S2 (for converting partial 3D hulls to a 3D hull stream), S3 (for tracking body parts in a 3D hull stream) and S4 (for behavioral recognition based on traces of body parts), manuals for the software and sample data. (ZIP) [file pone.0078460.s008.zip › Software S3 - 3DTracker/source files/3DTracker/res/play.bmp]

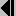

Supplement: File S1 — The file includes Software S1 (for recording partial 3D hulls), S2 (for converting partial 3D hulls to a 3D hull stream), S3 (for tracking body parts in a 3D hull stream) and S4 (for behavioral recognition based on traces of body parts), manuals for the software and sample data. (ZIP) [file pone.0078460.s008.zip › Software S3 - 3DTracker/source files/3DTracker/res/prev.bmp]
